# Home-Based Therapy After Stroke Using the Hand Spring Operated Movement Enhancer (HandSOME II)

**DOI:** 10.3389/fnbot.2021.773477

**Published:** 2021-12-17

**Authors:** Rafael Casas, Melissa Sandison, Diane Nichols, Kaelin Martin, Khue Phan, Tianyao Chen, Peter S. Lum

**Affiliations:** ^1^Biomedical Engineering, The Catholic University of America, Washington, DC, United States; ^2^MedStar National Rehabilitation Network, Washington, DC, United States

**Keywords:** exoskeleton, hand, neurorehabilitation, stroke, therapy

## Abstract

We have developed a passive and lightweight wearable hand exoskeleton (HandSOME II) that improves range of motion and functional task practice in laboratory testing. For this longitudinal study, we recruited 15 individuals with chronic stroke and asked them to use the device at home for 1.5 h per weekday for 8 weeks. Subjects visited the clinic once per week to report progress and troubleshoot problems. Subjects were then given the HandSOME II for the next 3 months, and asked to continue to use it, but without any scheduled contact with the project team. Clinical evaluations and biomechanical testing was performed before and after the 8 week intervention and at the 3 month followup. EEG measures were taken before and after the 8 weeks of training to examine any recovery associated brain reorganization. Ten subjects completed the study. After 8 weeks of training, functional ability (Action Research Arm Test), flexor tone (Modified Ashworth Test), and real world use of the impaired limb (Motor Activity Log) improved significantly (*p* < 0.05). Gains in real world use were retained at the 3-month followup (*p* = 0.005). At both post-training and followup time points, biomechanical testing found significant gains in finger ROM and hand displacement in a reaching task (*p* < 0.05). Baseline functional connectivity correlated with gains in motor function, while changes in EEG functional connectivity paralleled changes in motor recovery. HandSOME II is a low-cost, home-based intervention that elicits brain plasticity and can improve functional motor outcomes in the chronic stroke population.

## Introduction

Each year, stroke affects over 15 million people worldwide and 800,000 people in the US (Hankey, 2013; Benjamin et al., 2018). Stroke survivors are left with motor impairments that include hypertonia, inability to fully activate muscles, and abnormal inter-muscle coordination and muscle activation patterns (Kamper et al., 2003, 2006). These individuals tend to have long-term difficulty controlling movement of their impaired upper extremity and reintegrating their impaired hand into activities of daily living (ADL). Robotic therapy is effective in promoting motor recovery because it can apply precise and repetitive forces to the limb and allows completion of movements that would otherwise be impossible. Additionally, less effort by the user during activity based therapies (Dromerick et al., 2006) allows subject to fatigue slower, potentially enabling more effective training. Based on a meta-analysis of 45 clinical trials, robotic therapy was shown to produce larger gains in arm function, strength, and ADL ability than comparison interventions (Mehrholz et al., 2018). However, these functional gains might be too small to translate into meaningful clinical improvements for many patients. Additionally, the robotic technologies that have been tested in clinical trials have been mostly focused on shoulder and elbow recovery (Krebs et al., 2004; Staubli et al., 2009; Kung et al., 2010), with task practice that does not include object manipulation tasks (Lum et al., 2006a; Godfrey et al., 2013; Chen et al., 2021). Since hand use and object manipulation is the primary function of the upper extremity, task-specific training is required. Therefore, effective robotic devices should facilitate practice of complex multi-Degrees of Freedom (DOF) tasks involving the use of the hand to grasp and manipulate objects (Kwakkel and Kollen, 2007).

Table 1 provides a comparison of several powered and passive hand exoskeletons in terms of weight, cost and functions. Many powered hand robotic devices cannot be used during ADL because they are heavy, complex and often interfere with object manipulation tasks. Some powered hand devices (XGlove, PneuGlove, Hand of Hope, CyberGrasp, HandMATE) have task-specific training functionality and there have been examples of success in improving hand function of stroke patients when they can be integrated into ADL practice (Adamovich et al., 2009; Connelly et al., 2010; Susanto et al., 2015; Fischer et al., 2016; Sandison et al., 2020; Singh et al., 2021). Passive devices utilize springs or rubber bands to actuate, allowing devices to be lighter in weight and much less costly. The commercially available SaeboFlex, for example, has metal springs connected to the distal phalanx of each finger and can assist with tone management therapy and keeping the hand opened (Farrell et al., 2007). In the SaeboGlove, elastic bands provide extension assistance at the MCP and PIP joints (Saebo Inc., Charlotte NC). Another passive device, SCRIPT Passive Orthosis, uses SaeboFlex components and achieves greater finger ROM by applying external extension torques with a combination of passive leaf springs and elastic tension cords (Ates et al., 2013). These passive devices are better suited for home training, which enables increased ADL practice in real world environments. Home training has advantages because it reduces the need for therapist supervision and increases access to rehabilitation training, potentially increasing the dose available for highly motivated subjects (Lum et al., 2012). More practice and functional use of a stroke patient's affected limb offers more potential for clinical gains (Mehrholz et al., 2015). However, very few studies have explored the potential advantages of long-term home training studies (Nijenhuis et al., 2015, 2017; Chen et al., 2017).

**Table 1:** Comparison of exoskeletons for hand rehabilitation.

**Device**	**Weight**	**Cost**	**Functions/Differences**
XGlove	N/A	N/A	•Actuated device (Portable) •Mode 1-Cyclical stretching, Mode 2-Targeted extension assistance for active training •Glove-orthosis
PneuGlove	N/A	N/A	•Pneumatic glove (Tethered-not portable) •Independent extension assistance •Compatible with VR environment
Hand of Hope	0.7 kg (small/med) 0.8 kg (large)	N/A	•Linear actuated (Tethered) •Assists in hand opening or hand closing functional tasks •Paired with Games •EMG controlled
CyberGrasp	0.45 kg (+0.8 kg with CyberGlove)	N/A	•Powered (Tethered) •Fits around CyberGlove •Provides resistive force feedback to each finger to grasp computer generated models •Paired with Games
HandMATE	0.37 kg	N/A	•Actuated device (Portable) •Task specific training •Functional games •Feedback of movement
HandSOME II	0.25 kg	N/A	•Passive, assistive device (Portable) •Assists with finger extension using springs •Easy tension adjustment for 11 finger and thumb DOF
HandSOME I	0.22 kg	N/A	•Passive, assistive device (Portable) •Assists with hand opening extension using bands •Fingers and thumb coupled into 1 DOF.
SaeboGlove	N/A	$299	•Passive, assistive device (Portable) •Assists with finger extension at MCP, PIP, and DIP joints using various bands •Low profile, for mild flexor hypertonia
SaeboFlex	N/A	$599	•Passive, assistive device (Portable) •Assists with finger extension using one single spring for all finger PIPs and one for the thumb CMC •For moderate-severe flexor hypertonia

Due to the heterogeneous nature of stroke, patients demonstrate variance in response to treatment and long-term outcomes (Prabhakaran et al., 2008; Mozaffarian et al., 2016). Understanding this variability in responsiveness requires methods that investigate the neural mechanisms underpinning functional recovery and a method for optimal prescription of rehabilitative therapies. Functional connectivity (FC) measures the degree of integrated association between spatially separated brain regions responsible for executive motor function. Evidence suggests that measures which investigate the inter-regional complexity of brain networks are superior to behavioral measures such as baseline impairment (Riley et al., 2011; Wu et al., 2015) or neural measures of injury such as infarct volume and percent corticospinal tract injury (Wu et al., 2015). Resting state FC represents a means of investigating plasticity without confounding goal-directed neuronal action and external muscle outputs. It has also been shown to represent the engagement of the neural networks during motor tasks (Deco et al., 2011) and are predictive of subsequent motor performance (Hampson et al., 2006; Tambini et al., 2010; Wu et al., 2014). Research also indicates that FC measures at rest are biomarkers of cortical function post-stroke (Zhu et al., 2010; Rehme et al., 2011; Dubovik et al., 2012; Wu et al., 2015). Wu et al. followed 12 hemiparetic stroke patients undergoing a month of intensive therapy (Wu et al., 2015). Resting state surface EEG activity was recorded pre intervention, then at four time points throughout the intervention. The initial session indicated that connectivity with the ipsilesional primary motor cortex (M1) was a robust and specific marker of motor status, which accounted for 78% of the variance in motor function of the stroke patients. The study also indicated that connectivity with the ipsilesional M1 was a good biomarker of improvements in motor impairments, with the greatest improvements associated with the larger increases in ipsilesional M1 and premotor cortex (PM) connectivity.

Our lab previously developed the Hand Spring Operated Movement Enhancer (HandSOME I), a passive hand device that provides assistance to finger extension within a single gross grasp pattern (Brokaw et al., 2011). A 4-week longitudinal home training study was conducted with the device (Chen et al., 2017). Significant gains were seen immediately after training in tests of impairment and function. However, all of these gains were lost at a 3-month followup. The next iteration of the device, Handsome II, expands the HandSOME I concept, allowing customized adjustment of the extension assistance to 11 different finger joints and practice of more complex grasp patterns used in daily activities (Tianyao and Lum, 2016). In a prior study, we showed donning the device immediately increases extension range of motion in the fingers and improves ability in grasp and release tasks (Casas et al., 2021b). We also designed HandSOME II so that it could be produced at very low cost, so that a custom-built device could be given to patients in the study. The goal of this study was to determine the effectiveness of HandSOME II in an 8-week home training protocol. The study measured compliance with the prescribed training with a built-in sensor that collected data on movement repetitions performed and hours logged. Following the training, the device was given to the subject during a 3-month followup period. Clinical and biomechanical testing was performed all time points. We also used surface EEG to investigate changes in resting state FC and identify biomarkers associated with clinically defined impairments of sensorimotor control.

## Methods

The study enrolled 15 chronic stroke subjects. Inclusion criteria were: (1) diagnosis of stroke more than 6 months before enrollment; (2) ability to close the hand; (3) at least trace extension (opening) of the fingers; (4) adequate cognitive status (>24 on Mini Mental State Exam). The exclusion criteria were: (1) excessive pain in any joint of the affected extremity that could limit ability to cooperate with the protocols; (2) receiving oral or injected antispasticity medications during study treatment; (3) MCP and IP passive extension limit >30 degrees from full extension; (4) excessive tone in the fingers and thumb as determined by Ashworth scores ≥3; receiving physical or occupational therapy for the upper extremity.

### HandSOME II Intervention

**Figure 1:**
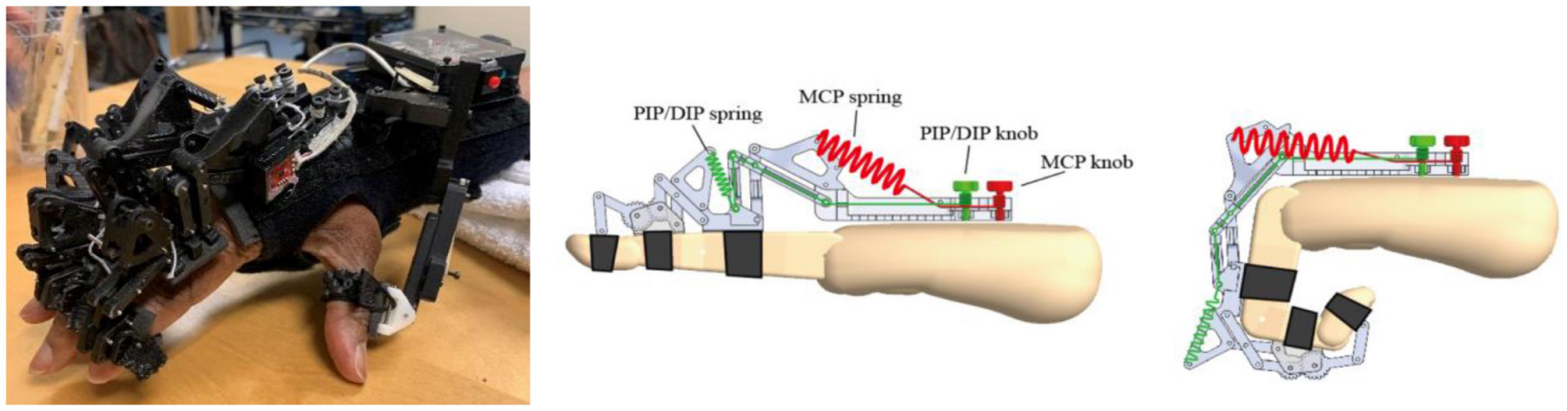
**(Left)** Spring driven HandSOME II with adjustable screws. **(Right)** Springs paths for MCP and PIP/DIP springs.

**Figure 2:**
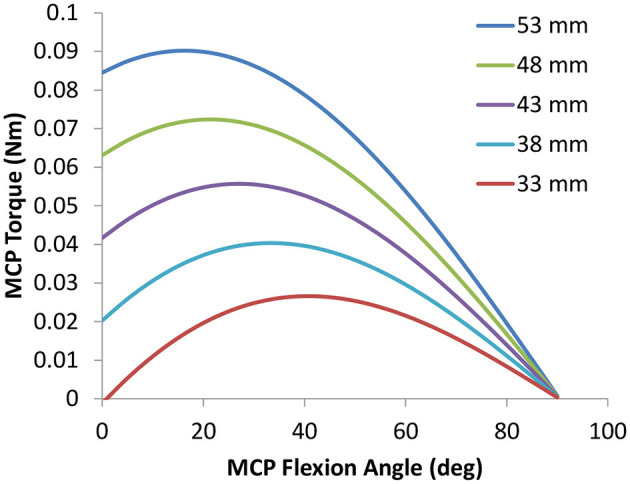
Typical Torque vs. Angle assistance curves for the MCP joint. The shape and peak torque change as the maximum spring length denoted in the legend is adjusted with the MCP knob. Similar profiles were applied to the PIP joint.

The HandSOME II is comprised of rigid mechanical linkages that allow isolated movement at almost all finger and thumb joints (Figure 1). For each of the four fingers, a linkage is strapped to the dorsal surface of each phalange. The linkage has centers of rotation that align with the metacarpophalangeal (MCP) and proximal interphalangeal (PIP) joints allowing free movement. All of the rigid parts are on the back of the fingers to allow hand closing into a fist when there is no space between fingers. Eight steel coil springs provide extension torque at the MCP and PIP joints of the 4 fingers (Figure 1). The operating length of each spring can be adjusted by knobs, and this changes the peak assistance torque applied. If the distal interphalangeal (DIP) also needs extension support, the linkage can be extended with a gear and finger cap. The gear rotates the PIP and DIP together in a fixed ratio and both joints are driven together by one spring. For the thumb, a different linkage is attached that has joints aligned with the carpometacarpal (CMC) joint for abduction/adduction and flex/extension. Elastic bands were used to support CMC abduction, CMC extension and interphalangeal (IP) extension (if needed). Figure 2 shows the change in the assistance torque profile as the adjustment knob is adjusted.

**Figure 3:**
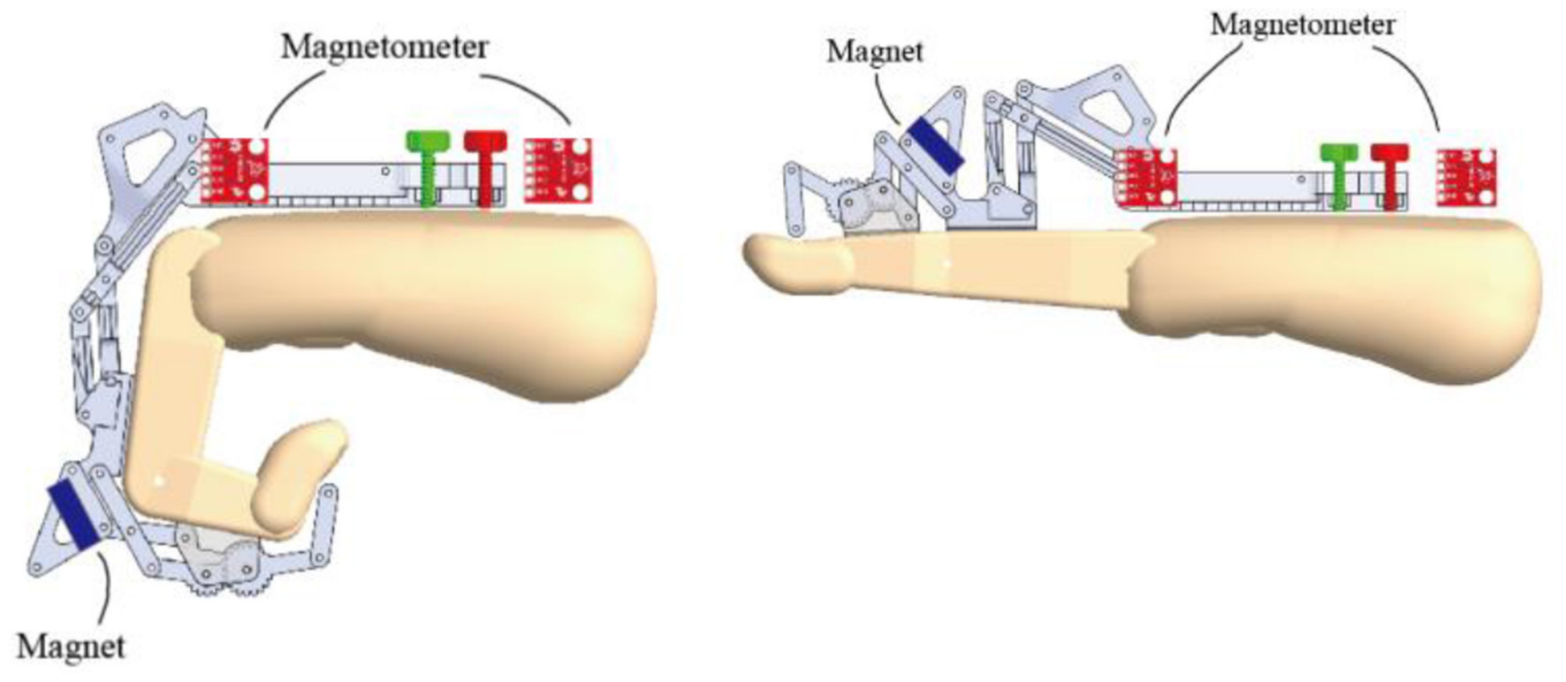
CAD drawing showing locations of magnetometers and magnet during finger extension and flexion. The magnet orientation is affected by both MCP and PIP rotation.

An activity tracker was fully integrated into the HandSOME II and collects index finger movement and the total time that the device was powered on (Figure 3). When the tracker is turned on, motion data is automatically stored on an SD card. The tracker consisted of a pair of magnetometers, a permanent disc-shaped magnet attached to the index finger linkage, and a microcontroller with integrated SD card located on the back of the hand. Movement of the index finger MCP or PIP rotated the magnet relative to the magnetometers on the back of the hand. One magnetometer was close to the magnet and the other was far enough away to be only minimally affected by the magnet. The difference in the two magnetometer signals was used to detect finger movements. Whole arm and wrist movements also affect the magnetometers due to earth's magnetic field, but the effect is the same for both units and is removed by using the difference in the magnetometer signals. The accuracy of the activity tracker was previously validated with healthy controls and stroke subjects (Casas et al., 2021a).

During their initial visit, engineers fitted the device to the subject's hand and finger lengths. Different options for small, medium and large phalange lengths were available. Spring tension was adjusted based on the therapist's assessment and patient feedback. Different stiffness springs were available to accommodate subjects with difference impairment levels. Only the minimum spring assistance was given in order to keep the fingers extended or to functionally open to grip objects. The subjects were instructed on how to don device and turn on the activity tracker before each of their home training sessions. Subjects were asked to perform a 90-min therapy session every weekday for 8 weeks, for a target of 60 h of wear time.

We chose a treatment intensity of 90-min sessions, five times a week because this was found to produce functional gains in our prior HandSOME study. We extended the treatment period from 4 weeks to 8 weeks in this study to try and produce more durable gains that were retained at followup. At each weekly visit to the lab, adjustments and repairs were made to the device, and the therapist prescribed several grasp and release tasks with various objects, based on performance and individual goals. Subjects were also encouraged to use the hand in everyday activities when wearing HandSOME II. The amount of movement practice during the weekly visits was kept at a minimum, so that any gains could be attributed to the home training. Engineers would collect data from the activity logger SD card and troubleshoot or repair any broken parts at the therapist's direction. Spring tension was also adjusted based on performance. At the end of 8 weeks, the sensor and logging electronics were removed and the HandSOME II was given to the subject. Subjects were encouraged to continue using the device during the 3-month follow-up, but there was no formal scheduled contact with the project team until the end of the follow-up period, when subjects were called in for evaluations. Subjects were instructed to contact the project team if their unit needed a repair. Additionally, subjects were asked to fill out a portion of a survey at 8 weeks and after the 3-month follow-up.

### Clinical Outcome Measures

Assessments were performed before and after the 8-week training intervention as well as 3 months after the training ended. Motor impairments at the shoulder, elbow, wrist, and fingers were assessed using the Fugl-Meyer (FM) assessment of the upper extremity (Fugl-Meyer et al., 1975). Reflexes, coordination patterns, and ability to perform several simple movements are tested in the FM. Functional use of the upper extremity was assessed using the Action Research Arm Test (ARAT) (Lang et al., 2006). Subjects were tested on their performance handling 19 items during Grasp, Grip, Pinch, and Gross movement. The Motor Activity Log (MAL) Amount of Use scale was used to assess the subjects' amount of limb use at home (Uswatte et al., 2006). In the structured interview, respondents were asked to rate their motor-impaired arm use during 30 ADL at home. Activities assessed included brushing teeth, buttoning a shirt or blouse, and eating with a fork or spoon. Hypertonia at the fingers, wrist and elbow was assessed using the Modified Ashworth Scale (MAS) (Bohannon and Smith, 1987). A flexor hypertonia score was calculated by averaging across flexor muscles at these joints, and an extensor hypertonia score was gotten by averaging across extensors. Grip Strength was quantified with a dynamometer (JAMAR 5030J1 Hand Dynamometer).

### Biomechanical Outcome Measures

**Figure 4:**
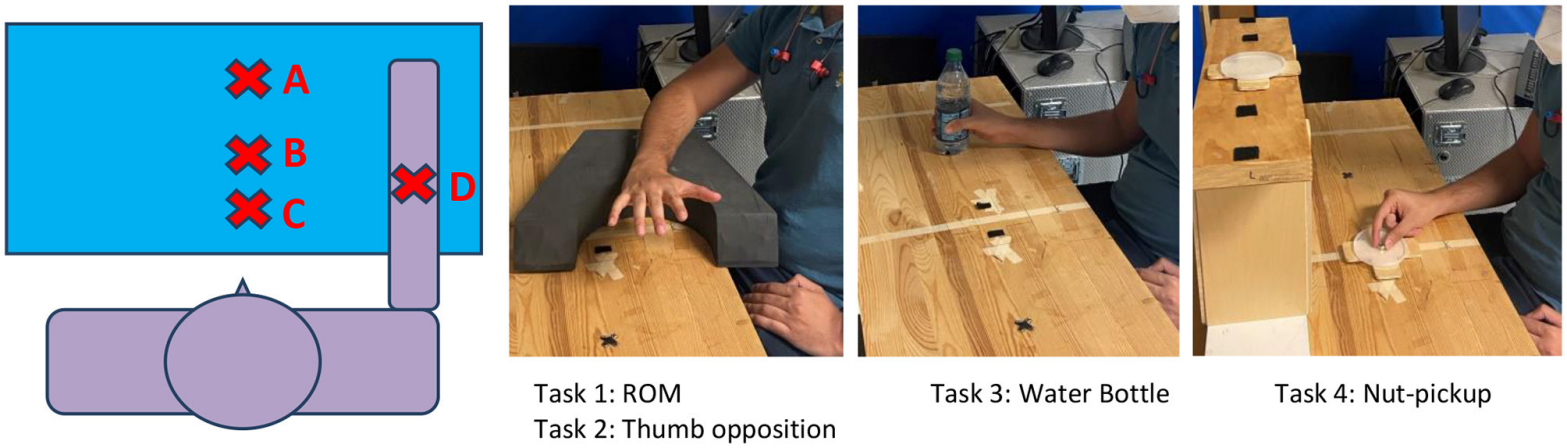
Subjects were seated in front of a testing table. Position A is the target location of the Nut-Pickup task, at shoulder height. Position B is the starting location of the nut. Position C is the start position for all tasks. Position D is the start location of the water bottle. The forward distances from the subject to positions B, C, and D were 11”, 6”, and 8” respectively. The forward distance to position A was determined by the length of the subject's outstretched arm at 90 deg of shoulder elevation and full elbow extension (at 100% of reach).

Subjects were seated in front of a standard testing table and performed the following tasks. Task #1–Digit range of motion (ROM): The forearm was supported on the table in a pronated position at midline. The task was to straighten fingers as much as possible from a closed fist position. Task #2–Thumb opposition: With the forearm in the same position as Task #1, the task was to touch the thumb tip to the tip of the 5th digit. Task #3–Water Bottle: Subjects were instructed to reach out and grasp a water bottle located laterally and elevate to the mouth to drink, then replace the bottle at the same location. Task #4–Nut pickup: Subjects picked up a M4 nut and placed it on a shoulder height shelf. The layout of the testing is described in detail in Figure 4.

Kinematic data was collected using the MiniBirds® (Ascension Technologies) electromagnetic motion capture system controlled by the Motion Monitor® Software (Innovative Sports Technology). Electromagnetic markers were taped at the nail of thumb, index, middle, and ring fingers. Markers were also placed on the back of hand, forearm proximal to the wrist and on the C7 vertebrae. The position and orientation of each marker had a sampling rate of 120 Hz. Raw data was exported into a custom Matlab program that calculated several metrics. Based on the Euler sequences recommended by the International Society of Biomechanics (Wu et al., 2005), several kinematic variables were calculated. The total flexion angle (0 represents full extension of the digit) was calculated for each distal phalange marker relative to the hand segment. This represents the sum of flexion/extension from all three joints of each digit. The smallest and largest flexion angle achieved was retained from each trial, presenting the furthest flexion and extension excursion. Finger ROM was calculated as the difference between the largest and smallest flexion angle. These angles were averaged across the four fingers prior to statistical analysis. Using the thumb distal tip marker, thumb abduction/adduction angles were also calculated yielding max and ROM values. For the reach and grasp tasks (3 and 4), trunk displacement was calculated from the farthest forward, lateral and vertical movements of the trunk coordinate frame relative to the starting point. A global measure of trunk movement was calculated by combining movement in these 3 directions using the Euclidean norm. Hand displacement was calculated similarly, except trunk movement in each direction was subtracted from hand movement first, so that the hand displacement metric was associated with arm movement only. Subjects attempted each task twice, with each trial capped at 40 s. The best metric of both trials of each task was reported.

### EEG

Eight of the 10 subjects received EEG evaluations at the pre and post-time points. EEG signals were recorded continuously from a 32 Ag/AgCl electrode cap. Data was sampled at 100 Hz and filtered using a bandpass of 0.1–40 Hz. Impedances of all electrodes were kept below 5KΩ. Three minutes of wakeful eye open resting state data was collected. Patients were seated with feet on the floor and instructed to look at a fixation cross. Preprocessing was performed in MATLAB, using the EEGLAB 14_1_2b tool following Makoto's pre-processing pipeline1 (UCSD, 2019). Bad channels and data segments were rejected using automated EEGLAB Artifact Subspace Reconstruction and confirmed visually. Removed channels were interpolated. The data was re-referenced to linked earlobes. Independent component analysis was performed on the continuous data, and artifacts were then removed using the machine learning Multiple Artifact Rejection Algorithm (Winkler et al., 2011). The primary outcome measure was resting state FC and was computed using magnitude-squared coherence (Coh_Rest_) (Steven Waterstone et al., 2020). The magnitude-squared coherence was computed using the formula:


$$\begin{array}{l} \text{Co}{\text{h}}_{\text{xy}}^{2}=\frac{|{\text{P}}_{\text{xy}}\left(\text{f}\right){|}^{2}}{{\text{P}}_{\text{xx}}\left(\text{f}\right)\cdot {\text{P}}_{\text{yy}}\left(\text{f}\right)} \end{array}$$

where the P_xx_(f) and P_yy_(f) were obtained via fast Fourier transformation and represent the power-spectrum density of signals x and y respectively; P_xy_(f) represents the cross-spectrum density, and Coh_xy_ is the frequency (f) dependent coherence coefficient of signals x and y.

Data was analyzed separately in the Alpha (8–12 Hz) and Beta (13–30 Hz) bands. Data from subjects with right hemisphere infarcts were flipped across the midline for subsequent analyses. The following four regions of interest overlying motor areas were then selected for analysis (Calabrò et al., 2019): electrodes overlying the ipsilesional premotor area (IF = F3, F7, FP3), homologous channels over the contralesional premotor area (CF), electrodes overlying the ipsilesional sensorimotor area (ICP = C3, CP3C, P3), and homologous channels over the contralesional sensorimotor area (CCP).

### Power and Data Analysis

The a priori power analysis was based on data from our previous pilot study with HandSOME I (Chen et al., 2017). The pre-post effect size for changes in the ARAT was dz = 1.05, with pre-post correlation *r* = 0.93. In order to detect an effect this large, when using a 2-tailed paired *t*-test with alpha = 0.05, a sample size of *N* = 16 is required for power >0.90. For the Fugl-Meyer, the pre-post effect size was dz = 1.13, with pre-post correlation *r* = 0.80. In order to detect this difference, *N* = 13 subjects are required for power >0.90. We were unable to reach this sample size, due to a slower than expected recruitment rate.

For each clinical, biomechanical and activity tracking outcome measure, outliers were removed with the detection method of z scores > 3. This was followed by a linear mixed model ANOVA with time points of pre-treatment, post-treatment and 3 month follow up. This was followed by paired sample *t*-tests to determine significant differences between: (1) baseline time point and 8 weeks post-training; (2) baseline time point and 3-month follow up. To determine if more intensive use of HandSOME produced better outcomes, for outcomes with significant gains, correlations were performed with the number of movements performed during training and the amount of wear time during training. For EEG analysis, paired sample *t*-tests were used to examine pre-post changes in COH_Rest_. Correlations were then computed between COH_Rest_ and behavioral variables. For this linear regression analysis, outliers were removed using the ROUT method (Motulsky and Brown, 2006), and data analyzed using ordinary least-squares regression. We applied a Bonferroni correction of 6 for chance significance to account for the multiple EEG variables.

## Results

Ten subjects completed the pre and post-outcome evaluations. Subject characteristics are shown in Table 2. Five subjects dropped out due to unrelated medical issues, pain when using the device, and lack of compliance with home training goals. Nine of the 10 subjects completed the post 3 month follow up. Home training compliance was also monitored with sensor data in nine of 10 subjects (technical issues w/1 patient). The amount of time the device was worn, and the number of movements varied considerably across all 10 subjects. Movement number ranged from a low of 3,178 movements to a high of 61,418 movements over the training period. The number of hours the device was used ranged from 33 to 134 h. There was no significant correlation between hours of training and movements performed (*r* = 0.474, *p* = 0.199). There was evidence that compliance was affected by impairment level, the correlation between number of movements and impairment level (baseline FM) was significant (*r* = 0.737, *p* = 0.023).

**Table 2:** Participant characteristics.

**Subject**	**Age**	**Sex**	**Stroke location**	**Stroke type**	**Chronicity (months)**	**Fugl-Meyer**	**ARAT**
1	69	M	Right BG, corona radiata	Ischemic	67	39	32
2	47	F	Left temporal and parietal lobes	Hemorrhage	45	37	22
3	53	F	Left MCA	Hemorrhage	61	23	4
4	63	M	Right pontine	Ischemic	13	47	39
5	60	M	Left MCA	Ischemic	86	30	26
6	38	M	Left basal ganglia	Infarct	18	40	37
7	49	F	Right lacunes of BG/corona radiata and parietal lobe	Infarct	11	54	47
8	66	M	Right MCA	Infarct	72	34	34
9	39	M	L basal ganglia	Infarct	7	45	27
10	71	F	Periventricular white matter (non-specific)	Ischemic	10	44	30

*MCA, middle cerebral artery; ARAT, Action Research Arm Test*.

Table 3 shows individual gains in the clinical outcomes and Table 4 summarizes the statistical analysis. Relative to baseline, mean FM scores increased by 2.8 ± 5.6 (*p* = 0.147) points after intervention and by 3.4 ± 4.6 (*p* = 0.055) points at the follow-up. ARAT scores increased significantly after intervention by 3.4 ± 4.5 (*p* = 0.039) but gains at the follow-up were no longer significant (1.1 ± 3.6, *p* = 0.375), as several subjects were unable to retain the gains achieved immediately after the intervention. Ashworth scores for flexors declined significantly after intervention (−0.21 ± 0.24, *p* = 0.022), but changes were not significant at the follow-up (−0.17 ± 0.32, *p* = 0.155). MAL scores improved significantly after intervention (0.58 ± 0.63, *p* = 0.018), and also at follow-up (0.70 ± 0.54, *p* = 0.005). When comparing the significant gains in clinical scores with amount of home training performed, several correlations were significant. Subjects who wore the device more had larger gains in the ARAT immediately after the intervention (*r* = 0.784, *p* = 0.012). The significant gains in the MAL immediately after treatment was strongly correlated with number of movements performed (*r* = 0.877, *p* = 0.002). The significant MAL gains at the follow-up time point was significantly correlated with wear time (*r* = 0.774, *p* = 0.014).

**Table 3:** Individual gains in outcome measures.

**#**	**FM**	**FM**	**ARAT**	**ARAT**	**MAL**	**MAL**	**ASH**	**ASH**	**Use (hours)**	**Movements**
	**post-pre**	**fu-pre**	**post-pre**	**fu-pre**	**post-pre**	**fu-pre**	**post-pre**	**fu-pre**		
1	8	8	1	−1	0.41	0.09	−0.38	0.00	79.8	14,118
2	−2	5	−4	−3	0.13	0.15	−0.25	−0.38	48.4	15,224
3	3	5	0	0	0.11	0.53	−0.25	−0.38	53.7	6,940
4	−3	−3	3	−1	0.51	0.40	−0.13	0.00	44.0	8,330
5	−3	−1	4	2	0.92	0.75	0.13	0.13	61.7	5,918
6	5	N/A	10	N/A	0.40	N/A	−0.38	N/A	33.3	3,178
7	8	2	10	0	2.20	1.34	−0.63	−0.75	84.4	61,418
8	13	9	4	4	0.14	0.83	−0.38	−0.38	65.7	8,590
9	−2	−2	0	0	0.20	0.51	0.13	0.25	42.5	N/A
10	1	8	6	9	0.81	1.74	0.00	0.00	134.0	22,804

*FM, Fugl-Meyer Test; ARAT, Action Research Arm Test; ASH, Modified Ashworth Test*.

**Table 4:** Mean change (SD) and statistical analysis of clinical outcomes.

	**Pre**		**Post**		**Followup**		**Time (p)**	**Post-pre (p)**	**Followup-pre (p)**
Fugl-Meyer	38.8	(9.0)	41.6	(10.5)	42.7	(9.3)	0.084	0.147	0.055
ARAT	27.2	(12.6)	30.6	(14.5)	30.1	(12.8)	0.051	0.039	0.375
Motor activity log	1.39	(0.61)	1.97	(0.80)	2.02	(0.84)	0.004	0.018	0.005
Ashworth-flexors	1.21	(0.51)	1.00	(0.57)	1.00	(0.61)	0.034	0.022	0.155
Ashworth-extensors	0.15	(0.26)	0.23	(0.27)	0.26	(0.30)	0.157	0.111	0.095
Grip strength (lbs)	29.7	(15.66)	29.8	(15.3)	33.22	(15.93)	0.43	0.90	0.31

A summary of the biomechanical analysis is presented in Table 5. In the range of motion task (Task #1), finger ROM increased significantly after the training period (19.8 ± 24.4 degrees, *p* = 0.031) and this gain was retained at the follow up (18.9 ± 10.3, *p* = 0.001). ROM gains were due to a combination of increased flexion and extension limits. Gains in flexion were 8.3 ± 20.2 degrees immediately after training and 5.2 ± 26.9 degrees at the followup. Finger extension gains were 12.1 ± 30.0 degrees after training and 13.0 ± 27.1 degrees at the followup. While each of these mean gains in flexion and extension limit were not significant individually, they combined to create significantly greater ROM immediately after training and also at the followup timepoint. In the water bottle task (Task 3), thumb abduction/adduction ROM increased significantly at the followup timepoint (33.6 ± 30.9 degrees, *p* = 0.011). In the nut pickup task (Task 4), hand displacement increased immediately after training (9.5 ± 13.0 cm, *p* = 0.046) and at the followup timepoint (12.7 ± 15.8 cm, *p* = 0.043). There were no other significant changes in the biomechanical measures. Additionally, there was no significant change to grip force performance.

**Table 5:** Summary of biomechanics data: mean (SD).

	**Pre**	**Post**	**Follow-up**	**Time (p)**	**Post-pre (p)**	**Followup-pre (p)**
**TASK 1**						
Finger flexion max (deg)	180.2 (19.5)	188.5 (19.6)	187.2 (14.0)	0.566	0.229	0.580
Finger extension deficit (deg)	50.3 (45.5)	38.2 (52.6)	29.6 (39.0)	0.323	0.233	0.189
Finger ROM (deg)	128.4 (51.0)	148.2 (51.7)	156.6 (43.4)	0.020	0.031	0.001
**TASK 2**						
Thumb cmc abd max (deg)	38.2 (16.9)	44.4 (18.5)	34.2 (10.0)	0.218	0.322	0.321
Thumb cmc add/abd ROM (deg)	25.4 (12.9)	37.9 (26.6)	33.0 (13.3)	0.338	0.216	0.311
**TASK 3**						
Finger extension deficit (deg)	34.7 (28.7)	28.4 (27.0)	26.9 (32.0)	0.564	0.362	0.636
Finger ROM (deg)	81.6 (21.9)	86.7 (20.1)	91.5 (33.0)	0.615	0.389	0.526
Thumb cmc abd max (deg)	45.0 (20.3)	49.1 (16.0)	42.5 (16.8)	0.280	0.240	0.506
Thumb cmc add/abd ROM (deg)	42.4 (18.1)	42.8 (13.2)	77.5 (36.3)	0.002	0.944	0.011
Hand displacement max (cm)	39.7 (8.5)	42.0 (9.8)	41.3 (5.1)	0.701	0.471	0.987
Trunk displacement max (cm)	13.0 (7.8)	12.1 (8.3)	9.3 (6.2)	0.350	0.740	0.169
**TASK 4**						
Hand displacement max (cm)	33.0 (14.7)	42.5 (6.8)	45.3 (8.6)	0.013	0.046	0.043
Trunk displacement max (cm)	13.0 (5.4)	13.5 (5.7)	12.5 (4.5)	0.945	0.800	0.770

**Figure 5:**
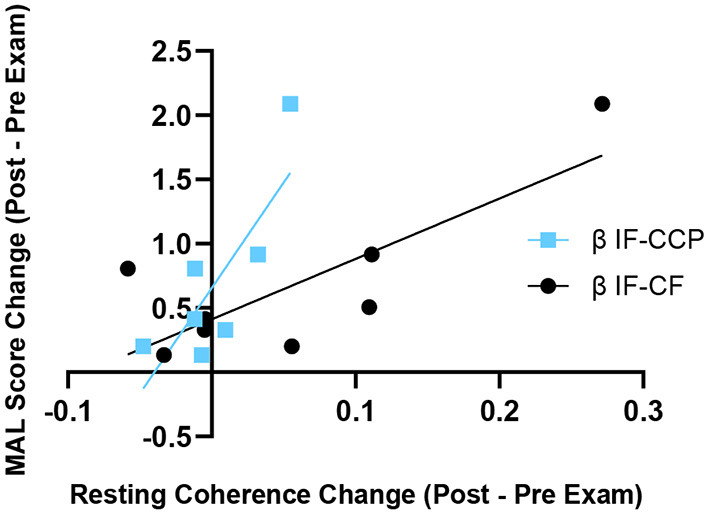
Change in resting state β coherence vs. change in MAL score, from pre to post-HandSOME II therapy. Greater increases in IF-CCP and IF-CF resting state coherence correlated with greater MAL gains (*p* = 0.006 and *R*^2^ = 0.92, and *p* = 0.108 and *R*^2^ = 0.64, respectively).

There were no significant changes in alpha or beta COH_Rest_ after 8 weeks of robotic hand rehabilitation in the eight subjects tested with EEG. However, correlation analysis revealed greater improvements in real life functional use of the upper extremity, as indicated in the MAL assessment, were correlated with greater increases in interhemispheric COH_Rest_ post-HandSOME II intervention (Figure 5). Specifically, increases in beta COH_Rest_ between the CCP and IF (*p* = 0.006, *R*^2^ = 0.92), were correlated with MAL gains. Changes in ARAT post-intervention did correlate with any changes in COH_Rest._

**Figure 6:**
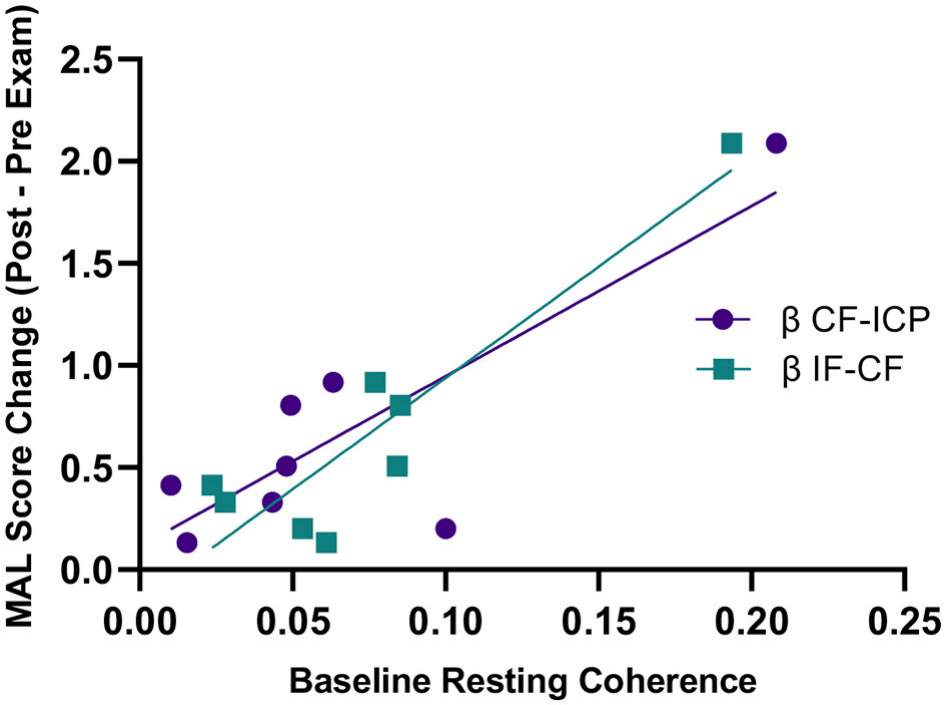
Baseline resting state coherence vs. change in MAL score from pre to post-HandSOME II therapy. Greater baseline β IF-CF and CF-ICP resting state coherence correlated with greater MAL gains (*p* = 0.012, *R*^2^ = 0.84 and *p* = 0.006, *R*^2^ = 0.70, respectively).

Higher baseline COH_Rest_ was a predictor for greater clinical gains in the MAL after 8 weeks of robotic hand therapy (Figure 6). Specifically, greater beta ICP and CF baseline COH_Rest_ (*p* = 0.006, *R*^2^ = 0.70), and CF and IF baseline COH_Rest_ (*p* = 0.012, *R*^2^ = 0.84), correlated with MAL gains. There was no correlation between baseline clinical scores in the ARAT and MAL, and the change in clinical scores post-HandSOME II home therapy.

As there was no significant change in FM score pre and post-intervention, correlational analysis was not performed on the FM scores.

We developed a survey to get design and device feedback at 8 weeks and at 3-month follow-up (Table 6). Responses were collected from 8 out of 10 subjects. The self-reported number of hours of weekly use of HandSOME II was 9.13 h during the 8-week training period but decreased to 5.16 h during the 3-month follow up. The respondents were generally positive about the device and 7 of 8 rated it 10/10 when asked if they would recommend it to another stroke patient. Six of eight respondents felt incorporation of games would improve the therapy routine.

**Table 6:** Survey results.

**Question**	**Response (# of votes)**	
Before the study, how many hrs/week did you work on upper extremity therapy?	4.75 (4.50) h	
During the study, how many hrs/week did you work on upper extremity therapy without the device?	4.75 (2.71) h	
During the study, how many hrs/week did you work on upper extremity therapy with the device?	9.13 (5.67) h	
What are the pros and cons about this HandSOME II device? What did you like/not like about it?	**Pros:**	**Cons:**
	•It helped •Disciplines you to be active; keeps hands open to be able to work •Got me exercising my hand and forearm •It helped me do a lot of things with my fingers •Improvement in strength of your hand •Helpful. •It highly improved my desire to exercise by right arm and hand.	•Breaking •Needs improvement to tie it on hand and make it easier to wear •Could be fragile •It needed to be serviced a lot •Making sure that the device is charged •No cons. •I am unable to articulate a negative aspect of HANDSOME. I loved and still love her!
What features would improve wearability (i.e., comfort and ease of use)?	•The thumb part •Improve the straps for someone to operate with one hand himself •Continuous finger sleeve •Tie my shoes •Making it easy to put on (the initial use) •Happy with how it was •House calls for minor adjustments by engineer	
What added features would improve your therapy routine?	•Regular calendar/email/text reminders for therapy exercises (3) •Log of therapy exercises (4) •Motion activity games (6) •Guided therapy with virtual reality (5) •Other feature: different objects (1)	
Would you recommend this device to other stroke patients?	Seven out of eight gave higher than a 10	
How much would you be willing to pay for the device?	$50–100 (4) $100–150 (2) $150–300 (2)	
Before this study, had you used the SAEBO? Have you continued to use it?	Yes (2)	
	No (6)	
During the 3 month period, how many hrs/week did you work on upper extremity therapy with the device?	5.16 (4.05) h	
During the 3 month period, you would have used the device more if—.	•Nothing to add •With a therapist/trainer •I swam a lot on my swimming pool •Complication-some of the straps broke •Felt not needed anymore •Used it when needed for chores •Time of the year–summer vacation, football just more time	

## Discussion

In this study, we saw users wearing the device in a range of 33–134 h over 8 weeks, with a wide range of total movements performed. Immediately after training, subjects had significant improvements in function (ARAT), flexor tone (Ashworth) and real-world limb use (MAL). At the 3-month followup, the MAL score changes continued to be significant. Biomechanical analysis was generally consistent with this clinical testing. Finger ROM and hand displacement in a reaching task were both improved immediately after training, with further gains during the followup period. EEG coherence analysis found pre-post changes that correlated with changes in self reported real world use (MAL). On average, subjects did not achieve the minimally clinically important difference (MCID) for the FM of about five points (Page et al., 2012; Klamroth-Marganska et al., 2014) and six points on the ARAT (Van Der Lee et al., 2001). However, there was a very large variance across subjects in responsiveness to treatment. When examining individual scores, five subjects achieved MCID on FM or ARAT at both post and follow up time points. MCID for the MAL has been set at 0.5 points by some by some authors (Van der Lee et al., 1999) and 1.0 by others (Lang et al., 2008). If using MCID of 0.5 points, four subjects achieved MCID post-treatment, and six achieved MCID at followup. When using the 1.0 MCID for the MAL, only one subject achieved MCID post-treatment, and 2 subjects achieved MCID at followup.

In the prior 4-week study with HandSOME I (Chen et al., 2017), the mean number of movements performed (both flexion and extension) was 8957 ± 13015 and total hours of use was 16.9 ± 11.3 h. In this study, we report 16280 ± 17938 total movements and 64.7 ± 29.3 h of use. Therefore, the dosage increased in terms of average number of movements and average use time compared with the HandSOME I study. *T*-test comparisons between studies found that wear time was significantly higher in this study (*p* < 0.001), but because of large variability across subjects, the number of movements were not significantly different. Comparison across studies might be compromised by different sensor technologies and differences between devices; HandSOME I constrains grasping movement to 1 DOF, while HandSOME II allows movement at 11 DOF, with the sensor only measuring movement at the index finger MCP and PIP. The HandSOME I study reported ARAT gains of 3.3 ± 2.6 and FM gains of 4.9 ± 4.1 immediately after training. Our study did not produce greater gains on these scales despite the increased dosage (ARAT gain of 3.4 ± 4.5, FM gain of 2.8 ± 5.6). However, the HandSOME II intervention did produce significant improvements in finger ROM, reaching extent and real-world limb use that were retained at the 3-month followup, while no significant effects were found at followup in the prior study. Therefore, it's possible the combination of a more advanced device and increased dosage were key factors in achieving gains in real-world use and finger control not achieved in the prior study. One possible explanation for the why the MAL gains were significant at followup, while the ARAT and FM were not, is the type of home training provided. Subjects were encouraged to use the device to manipulate objects in their home environment as part of the home training, with the more advanced HandSOME II enabling a large variety of grasp patterns. The MAL measures use of the impaired limb during ADL. It is possible that the home-based practice of ADL tasks using the exoskeleton, provides a more direct influence on real-world use of the limb (MAL) than impairment (FM) and functional gains (ARAT). We note that Constraint-Induced Therapy, which employs a forced home practice protocol using the impaired limb exclusively, produces larger gains on the MAL, and much smaller gains in impairment and function (Taub et al., 2013). Additional evidence supporting the importance of dose was found when correlating gains in outcomes to hours of wear and number of movements. Wear time was significantly correlated with ARAT gains post-treatment and with MAL scores at followup. Number of movements performed correlated significantly with MAL scores post-treatment. While number of movement repetitions is the likely dominant factor in driving recovery (Morris et al., 1997), wear time alone can be a positive influence as the springs hold the fingers in a more extended position (Salazar et al., 2019).

Our study showed that a home-based program using HandSOME II produces improvements in impairment and function within the range of improvements reported for robotic training performed in the clinic. Our subjects had gains of 2.8 Fugl-Meyer points after the intervention and 3.4 points at the follow-up. These gains are within the range of scores reported for other studies of robotic hand devices in chronic stroke. A review of prior studies found gains immediately after training ranging from 1.8 to 7.0 points on the FM scale (Farrell et al., 2007; Connelly et al., 2010; Stein et al., 2011; Fischer et al., 2016; Chen et al., 2017; Rowe et al., 2017; Calabrò et al., 2019; Kim et al., 2019). In studies that reported followup scores, FM gains ranged from 1.8 to 6.3 points (Connelly et al., 2010; Fischer et al., 2016; Rowe et al., 2017; Kim et al., 2019; Chen et al., 2021). All these studies were small scale, however this general trend in scores is consistent with the large multisite RATULS study that randomized 770 participants to 27 hours of hand and arm robotic therapy, 27 h of enhanced upper limb conventional therapy, or usual care. When robotic therapy was compared to usual care, participants in the robotic therapy group had an advantage of 2.79 Fugl-Meyer points after the intervention and 2.54 points at the followup 3 month. In terms of ARAT score, our subjects had gains of 3.4 points after the intervention and 1.1 points at followup. The RATULS study reported similar gains; ARAT gains were 1.37 points higher than usual care after treatment and 0.97 points higher at the 3 months followup. Smaller pilot studies have reported similar ARAT score changes (1.9–3.5) after robotic hand training (Fischer et al., 2016; Rowe et al., 2017).

While most of our insights in the 8-week training period relied on data from the activity logger, analysis of device use during the 3-month followup period was based solely on survey responses. The activity logger was removed from the HandSOME II following the 8-week training. Patients were asked to self-report activity time before, during, and after the study in the follow-up survey responses based on memory (Table 6). In the future, the survey should be completed at the initiation of the study and right after training. Also, in order to gather the most accurate data, the activity logger should be left on during the 3-month followup. Survey responses demonstrated that activity time dropped, in part, due to lack of 3rd party observation and inability to find time to use the device. However, subjects were generally positive about the device, with five patients reaching out for continued servicing of their device after followup.

Our results revealed subjects with the greatest improvements in real life functional use, as indicated by the MAL assessment, had the greatest increase in interhemispheric COH_Rest_ between the ipsilesional frontal and the contralesional centroparietal regions. Moreover, that subjects with the highest interhemispheric baseline COH_Rest_ (IF-CF and ICP-CF) made the greatest improvements in MAL after 8 weeks of HandSOME home therapy. These findings are consistent with previous animal and human studies that show low inter-hemispheric coherence is linked with more severe impairments in motor control and increases in inter-hemispheric sensorimotor coherence parallel recovery of motor control. One such study, Pellegrino et al. (2012) investigated interhemispheric resting state coherence of chronic stroke survivors undergoing 12 weeks of clinic based therapy with a motorized upper limb robotic device. They found the greatest increases in functional outcome in patients with greatest increases in interhemispheric primary M1 coherence. Opposing this, Wu et al. (2015) found no correlation between functional recovery of stroke patients undergoing intensive non-robotic therapy and interhemispheric M1-M1 coherence. Closer inspection of methodologies may explain these findings. Regions of interests (i.e., the electrodes chosen) are inconsistent throughout the literature making true comparison of studies difficult. For example, in two studies examining robotic therapy, Calabrò et al. (2019) grouped parietal and central electrodes, while Pellegrino et al. (2012) grouped central, parietal and frontal electrodes. Meanwhile, Wu et al. (2015) used a high density electrode system to investigate these as distinct regions, by taking M1 as the C3 electrode and calculating coherence with its six immediately surrounding leads.

Baseline clinical measures (MAL and ARAT) did not predict functional outcome after the 8 weeks of therapy. Similar results were found by Wu et al. (2015) who found baseline FM did not predict gains in stroke patient's motor performance after 4 weeks of intensive therapy. Additionally, they determined age, and MRI measures of infarct volume and percent of corticospinal tract injury did not predict functional upper limb outcome. Due to the heterogeneous nature of stroke there is high inter-subject variability in response to treatment (Bath et al., 2012; Saleh et al., 2012). Additionally, the vast and varied number of treatments under investigation suggests a need to not only quantify brain plasticity, but to investigate any biomarkers that can predict an individual's response to therapy in the hopes of optimizing therapy prescription. We have shown that EEG interhemispheric resting β state coherence is a robust neural marker for predicting recovery to home robotic therapy. Despite lower spatial resolution, EEG has additional benefits over other neuroimaging techniques, like MRI. For instance, it is suitable for patients with metal implants and an EEG can be administered bedside which may make it a more accessible biomarker.

In contrast with the MAL results, there was no correlation between changes in ARAT and changes in COH_Rest_, nor baseline COH_Rest_ and changes in ARAT score. The opposing results between the MAL and ARAT may result from the functionality of the HandSOME II device and the home therapy program. The high DOF assistance the HandSOME II provides was designed to enable practice of activities of daily living. As the MAL is a self-reported amount of usage, this measure may have been affected more by our intervention than the ARAT, which measures task completion of reach and grasp tasks in the clinic. The smaller treatment related gains in the ARAT compared to the MAL, may have affected the correlation analysis with EEG.

In a sample of 15 chronic stroke subjects, five withdrew from the study due to unrelated medical issues, pain when using the device, and lack of compliance with home training goals. A dropout rate of 33% is concerning and a larger scale study is needed to better identify the patient population who will tolerate this home-training protocol. Also, potential subjects must have some elbow and shoulder function to be able to use the HandSOME II in reach and grasp tasks. Importantly, the 10 subjects that completed the study were able to don the device without the help of a care giver. Devices were 3D printed and customized to individual's hand and finger sizes. Each week, engineers would repair device and do any necessary alterations during the weekly visit. This weekly interaction was likely an important component of the intervention in maintaining compliance, as self-reported wear time with the device decreased during the followup period, during which there were no scheduled interactions with project staff. The importance of the weekly interactions, suggest HandSOME II would be most effective when integrated into outpatient therapy. Alternatively, HandSOME II could be used independently without the need for weekly therapist supervision, if usability of the device can be improved, eliminating the need for frequent repairs, and training subjects to adjust the assistance levels on their own. We believe the main stimulus for gains was the 60 h of home training, and not the weekly visits to the clinic, which were focused on adjustment of the home training program and movement repetitions were kept to a minimum.

Recent studies have reported on the effectiveness of home-based approaches based on telerehabilitation strategies that require a remote therapist to motivate and supervise the home-training. Tele-AutoCITE is a home-based version of Constraint-Induced therapy that incorporates an instrumented workstation and remote supervision of the task practice (Lum et al., 2006b). In individuals with chronic stroke, Tele-AutoCITE produced gains in motor function and real world arm use that were not inferior to gains in a control group that received clinic-based Constraint-Induced Movement Therapy (Uswatte et al., 2021). A large multi-site study in subacute stroke delivered upper extremity tele-rehabilitation using 12 gaming input devices and videoconferencing software (Cramer et al., 2019). They found that this telerehabilitation approach was not inferior to dose-matched, in-clinic, one-on-one therapy. Both groups had reductions in motor impairment that were much larger than the MCID. Both of these telerehabilitation approaches rely on sensors that report on success or failure of task performance, but do not provide physical assistance during the training. In our protocol, common household objects were used for the task practice, under guidance from the therapist. It's possible using some aspects of telerehabilitation and integrating the sensor data from HandSOME II into a gaming interface would improve compliance.

## Conclusion

We developed a high-DOF exoskeleton that allows a large range of movement patterns and grasp types. The device allows a large range of motion that allows pointing, typing, key grip, power grasp and fine pinch. The device is very inexpensive to fabricate, allowing us to provide the subjects with a customized device during the followup period and after the end of the study. Our training protocol could be integrated with the outpatient phase of usual care and could potentially improve the rate and level of recovery of individuals after stroke.

## Data Availability Statement

The original contributions presented in the study are included in the article/supplementary material, further inquiries can be directed to the corresponding author.

## Ethics Statement

The studies involving human participants were reviewed and approved by MedStar Health Research Institute Institutional Review Board. The patients/participants provided their written informed consent to participate in this study.

## Author Contributions

PL, TC, MS, DN, and RC contributed to the conception and design of the study. PL and TC designed and modified the robotic device. RC, KM, and KP built each subject a custom device and attended all therapy sessions to evaluate the device. Therapy sessions were administered by DN, who was also responsible for subject recruitment. MS, TC, and PL collected, processed, and analyzed motion capture. RC and KM were responsible for design and analysis of magnetometer tracking data. MS and KP were responsible for EEG data collection, processing, and analysis. RC, PL, and MS wrote the first draft of the manuscript, but all authors contributed to manuscript revision, read, and approved the submitted version.

## Funding

This work was supported in part by the National Institutes of Health under Grant R21HD088783 and in part by the Department of Health and Human Services (Administration for Community Living, NIDILRR RERC) under Grant 90REGE0004. PL was also supported by the Professor Robert Meister Distinguished Faculty Fellowship.

## Conflict of Interest

The authors declare that the research was conducted in the absence of any commercial or financial relationships that could be construed as a potential conflict of interest.

## Publisher's Note

All claims expressed in this article are solely those of the authors and do not necessarily represent those of their affiliated organizations, or those of the publisher, the editors and the reviewers. Any product that may be evaluated in this article, or claim that may be made by its manufacturer, is not guaranteed or endorsed by the publisher.
